# Clinical response and changes in the fecal microbiota and metabolite levels after fecal microbiota transplantation in patients with inflammatory bowel disease and recurrent *Clostridioides difficile* infection

**DOI:** 10.20407/fmj.2020-021

**Published:** 2020-11-13

**Authors:** Hayato Osaki, Yasutaka Jodai, Keishi Koyama, Takafumi Omori, Noriyuki Horiguchi, Toshiaki Kamano, Kohei Funasaka, Mitsuo Nagasaka, Yoshihito Nakagawa, Tomoyuki Shibata, Naoki Ohmiya

**Affiliations:** Department of Gastroenterology, Fujita Health University, School of Medicine, Toyoake, Aichi, Japan

**Keywords:** Fecal microbiota transplantation, Short-chain fatty acid, Gut microbiota, Ulcerative colitis, Crohn’s disease

## Abstract

**Objectives:**

We determined the efficacy of fecal microbiota transplantation (FMT) and subsequent changes in fecal microbiota and short-chain fatty acid (SCFA) levels in patients with ulcerative colitis (UC), Crohn’s disease (CD), and recurrent *Clostridioides difficile* infection (rCDI).

**Methods:**

A filtered solution of Japanese donor feces was endoscopically administered. The efficacy of FMT was evaluated after 8 weeks using the Mayo score, Crohn’s Disease Activity Index (CDAI), and the absence of diarrhea with stool toxin negativity in patients with active UC, CD, and rCDI, respectively. For fecal microbiota analysis, the 16S ribosomal RNA gene was sequenced, and fecal SCFA levels were measured.

**Results:**

Clinical response was achieved in 5/20 (25%), 3/4 (75%), and 4/4 (100%) patients with UC, CD, and rCDI, respectively. Clinical remission was achieved in 4/20 (20%) and 1/4 (25%) patients with UC and CD, respectively. Linear discriminant analysis illustrated that UC responders had lower counts of *Clostridium* cluster XIVa before FMT and higher counts after FMT. Higher *Fusicatenibacter saccharivorans* counts in donors were significantly correlated with 8-week clinical remission. Patients with CD exhibited lower *Blautia*, *Dorea*, and *Eubacterium* counts before FMT and higher *Collinsella*, *Dorea*, and *Eubacterium* counts after FMT, accompanied by functional profiles predictive of SCFA fermentation and elevated fecal butyrate concentrations. Patients with rCDI displayed significantly lower abundances of *Clostridium* clusters IV and XIVa before FMT and higher abundances after FMT accompanied by elevated fecal propionate concentrations.

**Conclusions:**

FMT exhibited various efficacy against UC, CD, and rCDI by altering the gut microbiota and SCFA production.

## Introduction

Fecal microbiota transplantation (FMT) represents a breakthrough in the management of recurrent *Clostridioides difficile* infection (rCDI).^1^ FMT has also been used for patients with inflammatory bowel disease (IBD). In a randomized controlled trial of patients with active ulcerative colitis (UC), Moayyedi et al. reported that the 7-week remission rate was significantly higher in the FMT group than in the placebo group (24% vs. 5%).^2^ Paramsothy et al. reported that the 8-week rate of steroid-free clinical remission with endoscopic remission or response was significantly higher in the FMT group than in the placebo group (27% vs. 8%).^3^ Meanwhile, Costello et al. found that the 8-week steroid-free remission (32% vs. 9%), clinical response (55% vs. 23%), and clinical remission rates (47% vs. 17%) were significantly higher in the anaerobically prepared pooled-donor FMT group than in the autologous FMT placebo group,^4^ whereas Rossen et al. observed no significant difference in the 12-week response rate between the donor FMT and autologous FMT placebo groups (30.4% vs. 20.0%).^5^ Although no randomized controlled trials of FMT have been reported for Crohn’s disease (CD), some studies obtained 2- and 12-week response rates defined using the pediatric Crohn’s Disease Activity Index (CDAI) of 78 and 56%, respectively,^6^ and 6-, 12-, and 18-month clinical remission rates (Harvey–Bradshaw index ≤4) of 48, 32, and 23%, respectively.^7^

The levels of metabolites associated with gut microbiota, such as short-chain fatty acids (SCFAs) and bile acids, are also ameliorated after FMT. Seekatz et al. observed sustained increases in butyrate, acetate, and propionate levels and the recovery of secondary bile acids such as deoxycholate and lithocholate after FMT in patients with rCDI.^8^ Paramsothy et al. demonstrated an enrichment of *Eubacterium hallii* and *Roseburia inulinivorans* and increased levels of SCFA biosynthesis and secondary bile acids in patients with UC who achieved remission after FMT compared with the findings in patients who did not achieve remission after FMT.^9^

Japanese individuals have a unique traditional dietary culture that includes raw fish, fermented food, and seaweeds. The gut microbiome in Japanese individuals considerably differs from that of individuals in other countries in terms of the abundance of the phylum Actinobacteria, including *Bifidobacterium* and *Bacteroides plebeius*, which carry genes for seaweed-derived polysaccharide-degrading enzymes,^10^ and the depletion of the archaeon *Methanobrevibacter smithii*.^11^ Regarding gut microbial function in Japanese individuals, carbohydrate metabolism was overrepresented with concurrent decreases in replication, repair, and cell motility.^11^ In this regard, the microbial components of donor feces differ between Japanese individuals and individuals in other countries,^9,12^ possibly leading to discrepancies in the efficacy of FMT.

Thus, we prospectively determined the 8-week clinical response rates of single-dose FMT using fresh Japanese donor feces in 20, 4, and 4 patients with active UC, CD, and rCDI, respectively, and subsequent changes in the fecal microbiota, predictive functional profiles of microbial communities, and fecal metabolites such as SCFAs and bile acids.

## Methods

### Study design and patients

**Figure 1:**
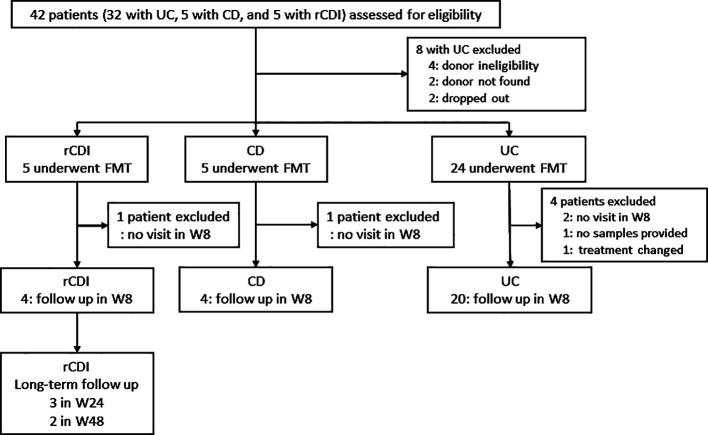
Study design. FMT: fecal microbiota transplantation, rCDI: recurrent *Clostridioides difficile* infection, CD: Crohn’s disease, UC: ulcerative colitis.

This single-center, prospective, open-label study evaluated the efficacy of FMT and subsequent changes in gut microbiota and fecal SCFA and bile acid concentrations in patients with rCDI, active UC, and CD. The study design is presented in Figure 1. Of consecutive patients who visited Fujita Health University Hospital between January 2016 and December 2017, 42 patients with rCDI, UC, or CD who hoped to undergo FMT were enrolled. The patients included subjects with CDI who experienced recurrence after more than one course of adequate antibiotics, such as metronidazole and/or vancomycin, and patients with UC or CD who did not achieve clinical remission or for whom remission was difficult to achieve with adequate treatment, including nutritional therapy, such as an elemental diet, 5-aminosalicylates, immunomodulators, corticosteroids, and/or biologics. Eligible patients with IBD had active UC with a Mayo score^13^ of ≥3 points or active CD with CDAI^14^ of 150 or higher. The exclusion criteria were as follows: allergy to the drugs used, pregnancy, bowel obstruction, gastrointestinal malignancies, admission to an intensive care unit, or a need for vasopressor therapy. The probiotics which the patients had already taken before FMT were not changed after FMT. Adverse events were monitored within 24 weeks after FMT. This study was reviewed and approved by the Institutional Review Board and Ethics Committee of the hospital. Informed consent was obtained from all patients. This study was registered with the University Hospital Medical Information Network (UMIN000020136). All authors had access to the study data, and they reviewed and approved the final manuscript.

### Donor selection

Donors (>20 years of age) were relatives, spouses, or acquaintances designated by the patients. All donors underwent psychological tests (Mini-International Neuropsychiatric Interview and Hamilton Depression Rating Scale); stool tests for parasites, glutamate dehydrogenase, and *C. difficile* toxin; stool culture; complete blood count and blood biochemical tests; cytomegalovirus antigenemia tests; tests for serum antibodies against human T-cell lymphotropic virus types 1 and 2, hepatitis B and C, and human immunodeficiency virus; tests for *Treponema pallidum*; a urea breath test to identify infection by *Helicobacter pylori*; esophagogastroduodenoscopy; and total colonoscopy. Pregnant women, subjects with positive results in the aforementioned tests, and patients with previous or current diabetes, depression, gastrointestinal malignancies, or infection were excluded.

### FMT procedure and sample collection

We instructed patients with IBD to take antibiotic pretreatment consisting of amoxicillin (1500 mg/day), fosfomycin (3000 mg/day), and metronidazole (750 mg/day) for 2 weeks until 2 days before FMT.^15^ However, if the patients denied this pretreatment because of fears of diarrhea, single-agent therapy with metronidazole (750 mg/day) was recommended for 1–2 weeks, whereas no antibiotic pretreatment was recommended for patients with rCDI. On the day of FMT, feces (approximately 100–150 g) were collected from donors, immediately diluted with twice the weight of sterile physiological saline, and stirred using a stomacher (i Mix^®^: Interlab, Osaka, Japan) to obtain approximately 250 ml of filtrate. Patients underwent standard bowel preparation with polyethylene glycol solution plus ascorbic acid (Moviprep, EA Pharma, Tokyo, Japan) in the morning on the day of FMT. In patients with rCDI or UC, fecal solution was infused into the cecum via colonoscopy, whereas in patients with CD, fecal solution was infused into the proximal jejunum via antegrade balloon-assisted enteroscopy without an overtube (Fujifilm Corporation, Tokyo, Japan). Donors’ feces used for FMT and recipients’ feces collected before antibiotic premedication and 8 weeks after FMT (and after 24 and 48 weeks in patients with rCDI), were stored at –80°C until analysis. The use of concomitant medications remained constant before and 8 weeks after FMT.

### Outcomes

The primary endpoint was clinical response 8 weeks after FMT. Clinical response was defined as the absence of diarrhea with a negative stool test for *C. difficile* toxin in patients with rCDI, a decrease from baseline in the total Mayo score of at least 3 points and at least 30% in patients with UC, and a decrease of CDAI of at least 70 points from baseline in patients with CD. Clinical remission was defined as a Mayo score of 2 or lower and no subscore higher than 1, and mucosal healing was defined as an endoscopic subscore of 0 or 1 and total CDAI of less than 150 in patients with UC and CD, respectively. Recipients were followed up at 2 and 8 weeks (and additionally at 24 and 48 weeks in patients with rCDI) after FMT for stool examination and blood tests and for confirmation of the clinical symptoms. Colonoscopy was performed again in patients with UC 8 weeks after FMT. The secondary endpoints were clinical remission 8 weeks after FMT, changes in fecal microbiota, SCFA and bile acid concentrations, and adverse events regarding FMT.

### DNA extractions and 16S rRNA gene-based sequencing

Fecal bacterial DNA was extracted using a Quick-DNA^TM^ Fecal/Soil Microbe Miniprep Kit (Zymo Research, Orange County, CA, USA). The V1-2 regions of the 16S rRNA gene were amplified using the 27fMOD and 338R primers, and then we performed high-throughput sequencing of this gene fragment on an Illumina MiSeq platform (2×250 base pair chemistry; Illumina, San Diego, CA, USA). Before analyzing the bacterial community, we removed sequences according to the following criteria: array reading start did not match the primer, quality score <20, <40 base pairs in length, and the corresponding pair sequence. The sequences that passed the quality filtering were subjected to chimera check using the EzBioCloud 16S database, and then raw sequences were analyzed using the QIIME 2.0 workflow script. Diversity analysis was performed using the diversity plug-in of QIIME 2.0 to analyze alpha and beta diversity. Linear discriminant analysis effect size (LEfSe version 1.0.7) was used to identify differentially abundant taxa within donor and recipient strains before and after FMT. The analysis was performed at the species level of the intestinal flora, and an effect size greater than 2 was the output. The alpha and beta diversity of the gut microbiota in donor and recipient feces were analyzed using the Shannon index and weighted UniFrac distance, respectively. For predictive functional profiling of microbial communities, we captured representative operational taxonomic unit sequences from the Greengenes database^16^ and performed a reconstruction of the metagenome using Phylogenetic Investigation of Communities by Reconstruction of Unobserved States (PICRUSt).^17^

### Fecal metabolite analysis

To measure fecal SCFA and bile acid concentrations, 100 mg of stool were weighed into a bead tube, a mixed solution of sodium acetate buffer and ethanol was added, and the sample was crushed and then thermally treated at 85°C for 30 min. The supernatant after centrifugation at 14,000 rpm for 10 min was diluted 4-fold with Milli-Q water, and the solid phase was extracted using a Bond Elute C18 cartridge (Agilent Technologies, Santa Clara, CA, USA). The obtained extract was dried, dissolved in 50% ethanol, and filtered through a hydrophilic PTFE filter with a pore size of 0.2 μm, and an internal standard solution (d4-CA, NDCA) was added. The fecal SCFA concentration in patients with UC, CD, and rCDI was measured by the postcolumn pH buffered electric conductivity detection method using high-performance liquid chromatography. The fecal bile acid concentration in patients with UC and CD was measured using liquid chromatography-quadrupole time-of-flight tandem mass spectrometry.

### Statistical analysis

Data are expressed as the median (range). For the comparison of gut microbiota diversity and bile acid and SCFA concentrations, Welch’s *t*-test and the Mann–Whitney U test were used appropriately after assessing normality using the Shapiro–Wilk test. Multivariate analysis was performed for 8-week clinical response and remission after FMT in the 20 patients with UC using unconditional logistic regression models after adjusting for age, sex, the duration and extent of disease, total Mayo scores before FMT, the use of antibiotic premedication, and the receipt of immunomodulators and anti-TNF antibody. Fisher’s R-to-Z transformation was conducted to compare correlations among the 8-week clinical response in patients with UC after FMT, age, disease duration, total Mayo scores before FMT, the use of antibiotic premedication, and the relative abundances of *Fusicatenibacter saccharivorans*, nonspecific *Eubacterium*, *Blautia obeum*, nonspecific *Dorea*, *B. massiliensis*, *D. formicigenerans*, *Ruthenibacterium*, *Ruminococcus bromii*, and *E. hallii* in donors and UC patients’ feces before antibiotic premedication and after FMT. Statistically significant differences in the relative abundance of taxa associated with groups of patients were examined using LEfSe).^18^ Functional profiles obtained using PICRUSt that were associated with differences in the predicted pathway abundances in patients before and 8 weeks after FMT were compared using the Mann–Whitney U test. *P*<0.05 indicated statistical significance.

## Results

### Patient characteristics and clinical outcomes

The clinical characteristics and FMT outcomes of patients with UC, CD, and rCDI are presented in Tables 1, 2, and 3, respectively. Eight-week clinical response was achieved in 5/20 (25%), 3/4 (75%), and 4/4 (100%) of patients with UC, CD, and rCDI, respectively. Eight-week clinical remission was achieved in 4/20 (20%) and 1/4 (25%) patients with UC and CD, respectively. The clinical response and remission rates in patients with UC were not significantly associated with age, sex, the duration or extent of disease, total Mayo scores before FMT, and the use of antibiotic premedication, immunomodulators, or anti-TNF antibody (*P*=0.277, 0.145, 0.220, 0.352, 0.399, 0.852, 0.999, and 0.999; 0.990, 0.985, 0.985, 0987, 0.990, 0.995, 0.986, and 0.994, respectively).

### Adverse events

There were no serious adverse events, such as fever, abdominal pain, sepsis, or perforation, in any patients within 24 weeks after FMT.

### Fecal microbiota analysis

#### Microbial composition of donors and patients’ feces

The relative abundances (%) of the genera and some species of the fecal microbiomes of 28 donors, 5 responders with UC, 15 nonresponders with UC, 4 patients with CD, and 4 patients with rCDI in the present study in comparison with previous Japanese data reported by Nishijima et al.^11^ are presented in the Supplemental Table.

### Alpha and beta diversity

**Figure 2:**
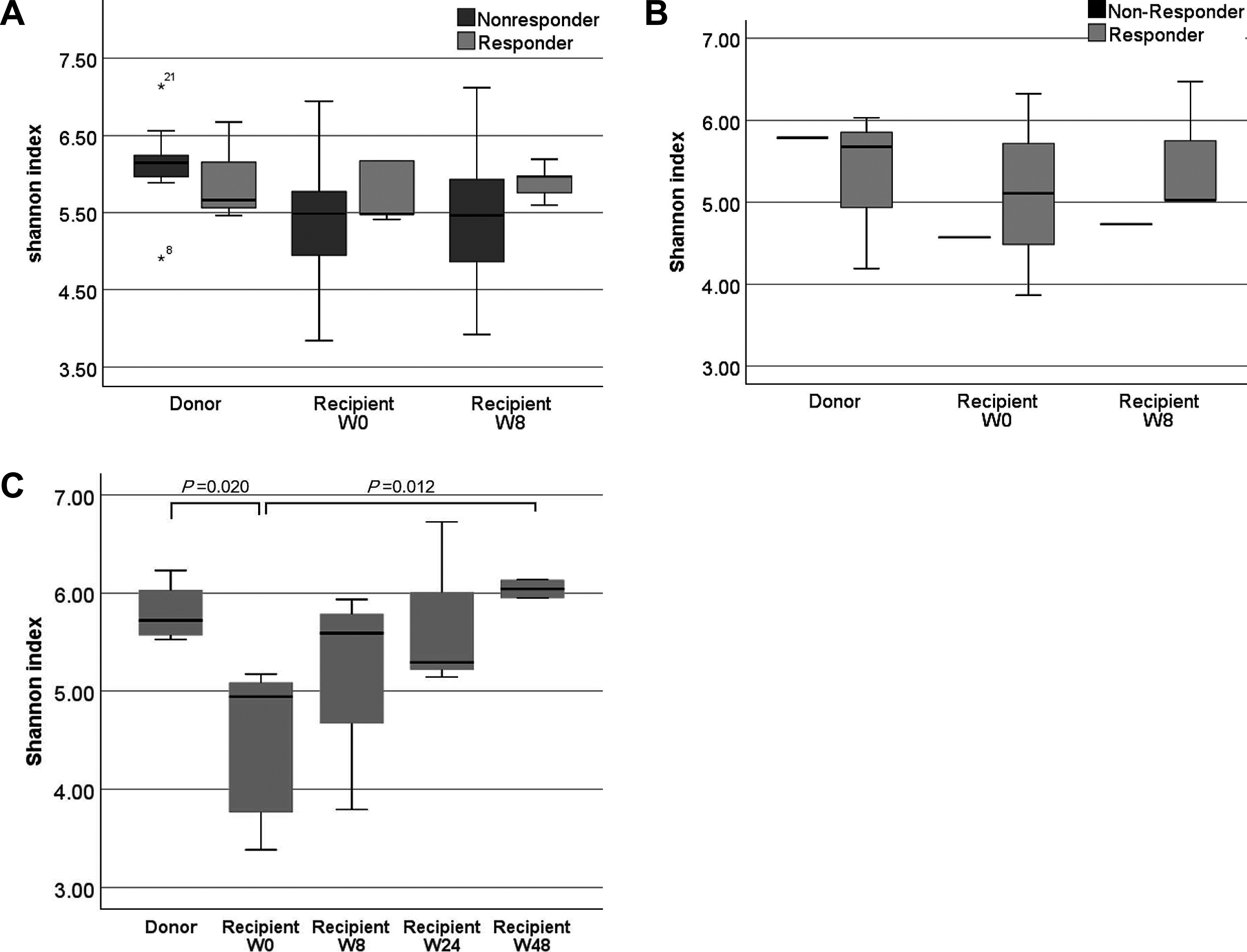
Fecal microbiota alpha diversity (Shannon index) in patients before and after fecal microbiota transplantation compared with that in healthy donors. The ends of the box represent the upper and lower quartiles, the horizontal line inside the box represents the median, and the whiskers represent the highest and lowest levels. Statistical analysis was performed using the Mann–Whitney U test. A. Ulcerative colitis. B. Crohn’s disease. C. Recurrent *Clostridioides difficile* infection.

Alpha diversity before antibiotic premedication and FMT in nonresponders with UC was significantly lower than that in donors (*P*=0.002) and remained lower at 8 weeks after FMT (*P*=0.003), but no significant difference was observed in responders with UC (Figure 2A). Beta diversity was significantly different between nonresponders and responders at 8 weeks after FMT (*P*<0.001). In patients with CD, there were no significant changes in either alpha or beta diversity (Figure 2B). Alpha diversity before FMT in patients with rCDI was significantly lower than that in donors (*P*=0.020), but it gradually increased, eventually becoming significantly higher at 48 weeks after FMT compared with that before FMT (*P*=0.012, Figure 2C). Beta diversity also became close to that of donors 8 weeks after FMT (*P*=0.005).

### LEfSe

Among patients with UC, five responders exhibited a lower abundance of *Clostridium* cluster XIVa, including *F. saccharivorans* and *Eubacterium*, before FMT than donors (Figure 3A), but no significant difference in the abundance of these bacteria was observed at 8 weeks after FMT (Figure 3B). Responders displayed enrichment of Lactobacillales and *Streptococcus* before antibiotic pretreatment and FMT relative to donors, which was possibly associated with probiotic intake (Figure 3A). Responders exhibited higher abundances of *Clostridium* cluster XIVa, including *B. obeum*, *Dorea*, and *Eubacterium*, and *Clostridium* cluster IV, including *Ruthenibacterium*, at 8 weeks after FMT than before antibiotic pretreatment (Figure 3C). In patients with UC, only a higher abundance of *F. saccharivorans* among donors was significantly correlated with 8-week clinical remission after FMT (correlation coefficient=0.579, *P*=0.0064). Nonresponders featured a lower abundance of many Clostridiales, including Lachnospiraceae, Eubacteriaceae, and Ruminococcaceae, before antibiotic pretreatment and FMT than donors, and these differences remained significant at 8 weeks after FMT (Supplementary Figure 1A–C).

Patients with CD exhibited a lower abundance of *Clostridium* cluster XIVa, including *Blautia*, *Dorea*, and *Eubacterium*, before FMT than donors (Figure 3D). They exhibited higher abundances of *Clostridium* and *Parvimonas micra* at 8 weeks after FMT than donors (Figure 3E). They also displayed significant enrichment of *Collinsella*, *Dorea*, and *Eubacterium* at 8 weeks after FMT compared with that before FMT (Figure 3F).

Patients with rCDI displayed significantly lower abundances of *Clostridium* cluster XIVa, including *F. saccharivorans*, *C. clostridioforme*, *Agathobaculum*, *B. caecimuris*, and *B. faecis*; *Clostridium* cluster XIVb, including *Anaerotignum*; *Clostridium* cluster IV, including *Faecalibacterium*; and *Clostridium* cluster IX, including *Sellimonas intestinalis*, before FMT than donors (Figure 3G), and significantly increased abundances of *Clostridium* cluster IX, including *Sellimonas* and *Oscillibacter*; *Clostridium* cluster XIVb, including *Anaerotignum*; and *B. thetaiotaomicron* were observed at 8 weeks after FMT (Figure 3H). The increased abundances of *Clostridium* cluster IX and XIVb were maintained until 24 weeks after FMT (Figure 3I). At 48 weeks after FMT, there were no significant differences because of the small sample sizes. The enrichment of *C. butyricum* in patients with rCDI was possibly attributable to probiotic intake.

A summary of gut microbiota and its changes following FMT are presented in Table 4.

### PICRUSt

Statistically significant changes in predicted pathway abundances before and 8 weeks after FMT in patients with CD and rCDI are presented in Supplementary Figure 2A and B, respectively. Patients with CD exhibited increased predicted abundances of bacteria associated with SCFA fermentation. Patients with rCDI featured increases in predicted abundances of microbes associated with the biosynthesis of cobalamin, methionine, pantothenate, coenzyme A, and thiamin, etc. Neither responders nor nonresponders with UC displayed significantly increased predicted abundances.

### Fecal SCFA levels

**Figure 4:**
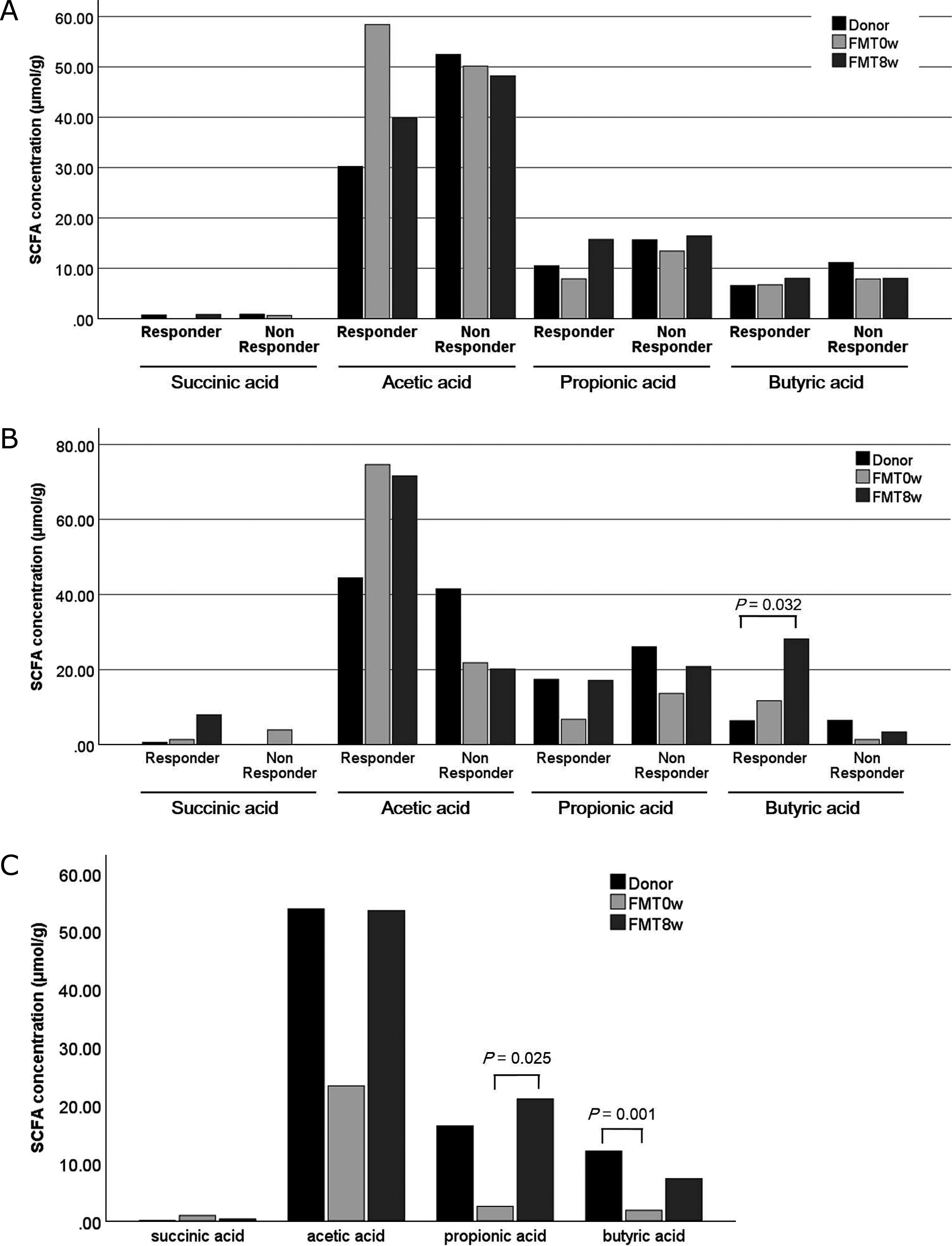
Fecal short-chain fatty acid concentrations. Statistical analysis was performed using the Mann–Whitney U test. A. Ulcerative colitis. B. Crohn’s disease. C. Recurrent *Clostridioides difficile* infection.

In patients with UC, neither responders nor nonresponders exhibited significant changes in fecal SCFA levels compared with those in donors or between before and after FMT (Figure 4A). In patients with CD, responders had a significant increase in fecal butyric acid content at 8 weeks after FMT compared with that in donors (*P*=0.032, Figure 4B). Patients with rCDI had a significantly lower level of fecal butyric acid before FMT than donors (*P*=0.001). Fecal acetic acid and propionic acid levels were also reduced, but the differences were not significant (*P*=0.129 and 0.068, respectively). Fecal propionic acid levels were increased significantly (*P*=0.025) at 8 weeks after FMT compared with those before FMT. Acetic acid and butyric acid levels were also increased, but the differences were not significant (*P*=0.233 and 0.107, respectively, Figure 4C).

### Fecal bile acid levels

In responders with UC, there were no significant differences in fecal primary or secondary bile acid levels between donors and patients before FMT, nor were there significant differences in patients between before and 8 weeks after FMT. In nonresponders with UC, the levels of secondary bile acids, including deoxycholic, lithocholic, and ursodeoxycholic acids, were significantly lower in patients both before and 8 weeks after FMT than in donors (Supplementary Figure 3A). In patients with CD, there were no significant differences in fecal primary or secondary bile acid levels between donors and patients before FMT, nor were there significant differences between patients before and 8 weeks after FMT (Supplementary Figure 3B).

## Discussion

The present study demonstrated that the 8-week clinical response and remission rates of single-dose FMT using Japanese-donor fresh feces for patients with UC were 25 and 20%, respectively, which were similar to the results of previous randomized controlled trials. The efficacy of FMT for UC remains controversial, but in most trials, procedures repeated 6–40 times achieved significant clinical responses and/or remission.^2–4^

Regarding FMT for Japanese patients with UC, Nishida et al. reported that the proportion of *Bifidobacterium* was significantly higher in the donor feces used for responders than in that used for nonresponders, whereas the proportions of *Lactobacillales* and *Clostridium* clusters IV and XI were significantly lower.^19^ Ishikawa et al. found that combination therapy with FMT and antibiotics for UC resulted in higher response rates than FMT alone, which was correlated with the recovery rate of Bacteroidetes that was reduced by antibiotics.^20^ The Japanese patients with UC in this study had significantly lower alpha microbial diversity including the depletion of *Clostridium* cluster XIVa than donors, but at 8 weeks after FMT, this depletion was diminished in responders but not in nonresponders. Furthermore, the abundance of *Clostridium* cluster XIVa was significantly increased in responders at 8 weeks after FMT compared with that before FMT. In these bacteria, however, only the higher abundance of *F. saccharivorans* in donors was significantly correlated with 8-week clinical remission after FMT (correlation coefficient=0.579, *P*=0.0064). *F. saccharivorans* was isolated from human feces by Japanese scientists and was characterized by the production of lactic acid, formic acid, acetic acid, and succinic acid as fermentation end-products from glucose.^21^ It has been reported that the fecal counts of these bacteria were lower in Japanese patients with active UC than in patients with quiescent UC and healthy controls, and these changes were associated with the induction of interleukin 10 production by lamina propria mononuclear cells in patients with UC.^22^ *Clostridium* clusters IV and XIVa, including *F. saccharivorans*, are high butyrate-producing species, and these bacteria play an anti-inflammatory role.^23^ To investigate the relationship between the efficacy of FMT and the metabolites of these bacteria, fecal SCFA and bile acid concentrations were measured. However, there were no significant differences in their levels between before and after FMT in patients with UC.

Next, for patients with CD, the 8-week clinical response and remission rates of single-dose FMT using fresh Japanese donor feces were 75% and 25%, respectively, in this study. Although randomized controlled trials have not yet been reported, the efficacy of FMT against CD is controversial. Gutin et al. stated that 3 of 10 patients (30%) with CD achieved a clinical response using frozen material^24^, whereas He et al. found that 17/25 (68%) and 12/25 (52%) patients with CD achieved clinical remission and response, respectively, using fresh donor material delivered into the distal duodenum via gastroscopy.^7^ We also performed FMT via the infusion of fresh donor material into the proximal jejunum via antegrade enteroscopy and demonstrated similar efficacy as He et al. Intriguingly, unlike UC, CD was associated with low abundances of *Clostridium* clusters IV and XIVa, followed by the restoration of these bacteria, increased fecal butyrate levels, and eventually amelioration of the changes of CDAI by FMT in the present study. Many previous studies reported that *Faecalibacterium prausnitzii* has a lower abundance in CD, but this was not detected by LEfSe in this study.^25,26^

FMT is a highly effective and robust therapy for rCDI that increases gut bacterial diversity including increased counts of Bacteroidetes species and *Clostridium* clusters IV and XIVa and decreased counts of Proteobacteria species.^1^ LEfSe in the present study revealed significantly lower abundances of many SCFA-producing bacteria in addition to *Faecalibacterium* before FMT and increased abundances of *B. thetaiotaomicron* and some of the aforementioned bacteria accompanied by changes in fecal SCFA levels after FMT, in line with the results of previous studies.

Finally, although bacterial diversity was not significantly increased by FMT in patients with IBD, some patients achieved clinical responses. In these patients, although the bacterial diversity was unchanged by FMT, gut microbial component was changed. Symptoms may have been improved with increased abundances of bacteria such as butyric acid-producing bacteria. In this study, because defecated stool was used as the sample, SCFA and bile acid concentrations may differ from the actual intestinal environment. Therefore, the relationship of changes in SCFA and bile acid concentrations with the efficacy of FMT remains controversial.

This study had some limitations, such as its small sample size and open-label design using different donors selected by the patients. The gut microbiota of healthy subjects greatly differs among individuals, and the efficacy of FMT and gut microbiota changes induced by this treatment may be affected by differences in the feces used.

In conclusion, FMT using fresh Japanese donor feces was effective against IBD and rCDI by altering gut microbiota and SCFA production. The newly detected bacteria associated with an improvement of the disease pathology in the present study may be good bacterial targets. Further studies using these bacteria in larger and other ethnic series samples are needed.

## Supplementary Material

Supplementary Figures

Supplementary Table

## Figures and Tables

**Figure 3:**
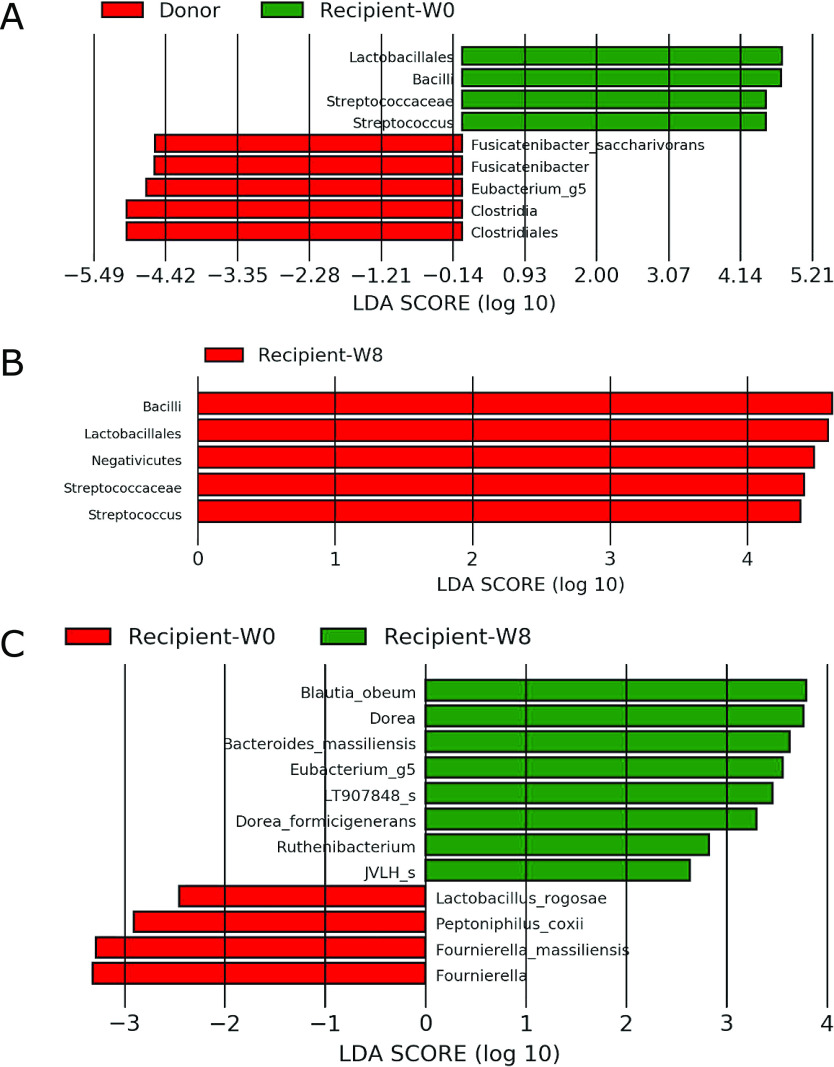
Linear discriminant analysis effect size. The analysis was performed using LEfSe version 1.0.7. A. Responders with ulcerative colitis before fecal microbiota transplantation compared with donors (n=5). B. Responders with ulcerative colitis at 8 weeks after fecal microbiota transplantation compared with donors (n=5). C. Comparison of ulcerative colitis responders between before and 8 weeks after fecal microbiota transplantation (n=5).

**Figure 3:**
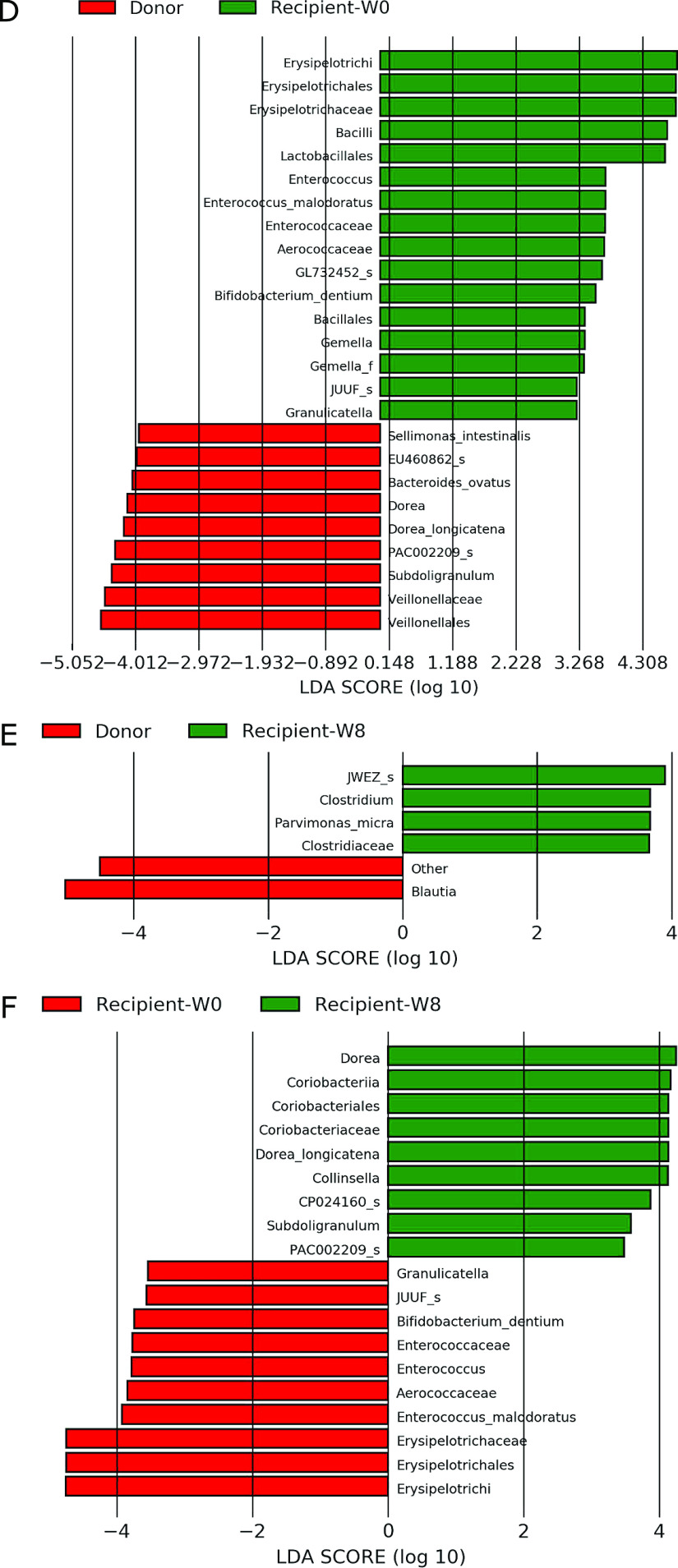
continued D. Patients with Crohn’s disease before fecal microbiota transplantation compared with donors (n=4). E. Patients with Crohn’s disease 8 weeks after fecal microbiota transplantation compared with donors (n=4). F. Comparison of patients with Crohn’s disease between before and 8 weeks after fecal microbiota transplantation (n=4).

**Figure 3:**
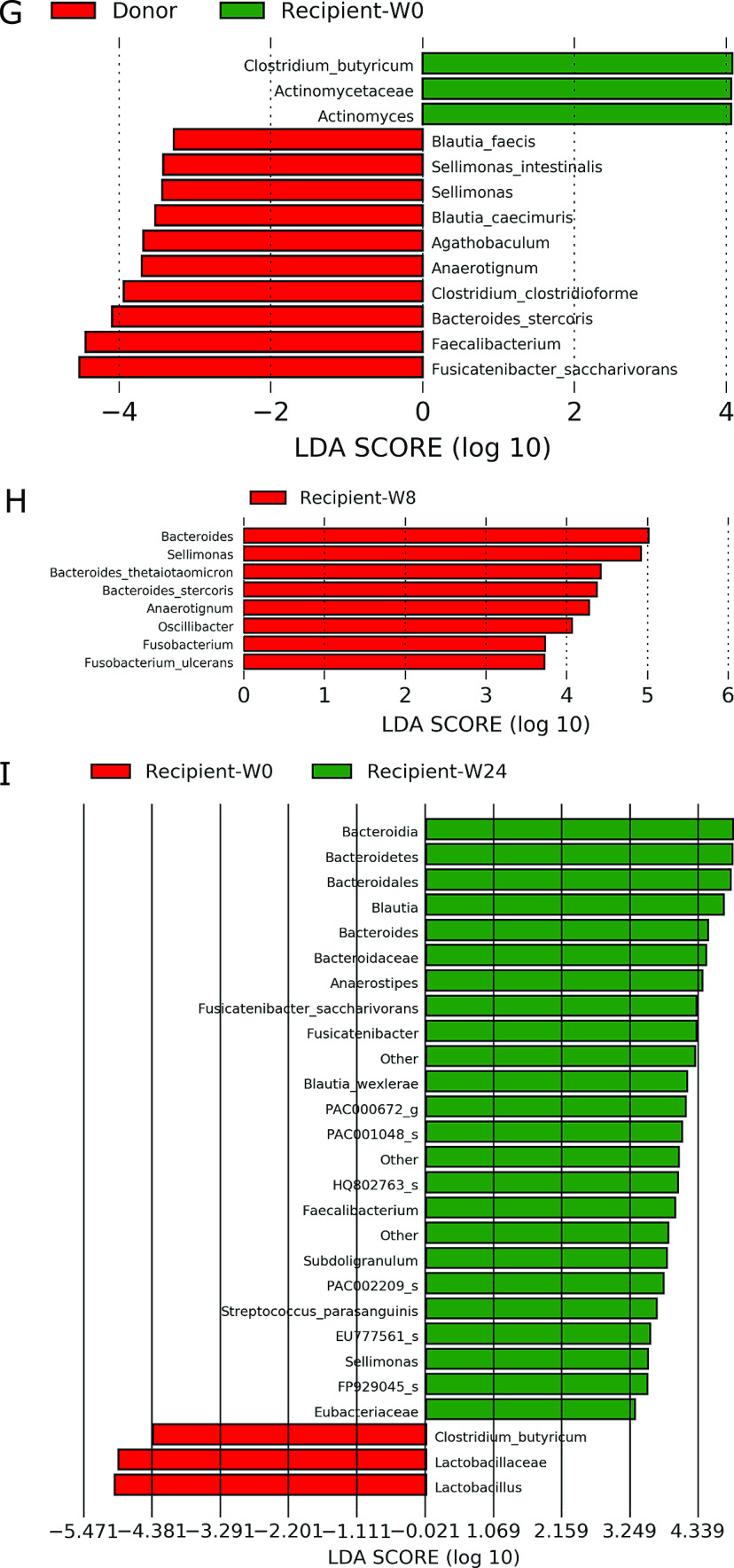
continued G. Patients with recurrent *Clostridioides difficile* before fecal microbiota transplantation compared with donors (n=4). H. Comparison of patients with recurrent *Clostridioides difficile* between before and 8 weeks after fecal microbiota transplantation (n=4). I. Comparison of patients with recurrent *Clostridioides difficile* between before and 24 weeks after fecal microbiota transplantation (n=3).

**Table1:** Clinical characteristics and outcomes after fecal microbiota transplantation in patients with ulcerative colitis

#	Sex	Age (y)	Duration (y)	Extent	Concomitant medications					Antibiotic pretreatment	Total Mayo score				Partial Mayo score			Endoscopic Mayo score	
					5-Aminosalicylates	Glucocorticoids	Immunomodulators	Tumor necrosis factor antagonists	Probiotics		preFMT	postFMT	Δ	Outcome	preFMT	postFMT		preFMT	postFMT
												8W				2W	8W		8W
Responder																			
1	M	56	16	Pancolitis	+	+	−	−	Bio-Three	−	5	2	3	Clinical response	3	0	0	2	2
2	M	46	12	Pancolitis	+	−	−	−	−	+	4	0	4	Clinical remission	3	0	0	1	0
3	F	62	15	Proctitis	+	−	−	−	Miya-BM	+	4	0	4	Clinical remission	3	3	0	1	0
4	M	43	10	Pancolitis	+	−	−	−	Bio-Three	+	5	1	4	Clinical remission	5	2	1	0	0
5	F	24	9	Left-sided colitis	+	+	−	−	Bio-Three	−	4	1	3	Clinical remission	2	2	0	2	1
Nonresponder																		
6	F	59	10	Left-sided colitis	+	+	−	−	Bio-Three	−	7	8	1	Nonresponse	5	6	6	2	2
7	M	20	2	Left-sided colitis	+	+	Azathioprine	−	Bio-Three	−	5	5	0	Nonresponse	3	3	3	2	2
8	F	57	7	Pancolitis	+	+	−	−	Miya-BM, Biofermin	−	10	8	2	Nonresponse	8	12	6	2	2
9	M	37	19	Left-sided colitis	+	−	−	−	−	−	3	4	1	Nonresponse	2	1	3	1	1
10	M	44	27	Left-sided colitis	+	−	Mercaptopurine	Infliximab	−	−	3	3	0	Nonresponse	2	3	2	1	1
11	M	33	11	Pancolitis	+	−	Azathioprine	Infliximab	−	+	4	4	0	Nonresponse	2	2	3	2	1
12	F	22	3	Pancolitis	−	−	−	Infliximab	Biofermin	+	8	9	1	Nonresponse	6	10	7	2	2
13	F	28	4	Pancolitis	+	−	−	−	−	+	6	7	1	Nonresponse	4	7	5	2	2
14	M	27	6	Pancolitis	+	−	Azathioprine	−	−	+	6	5	1	Nonresponse	4	3	3	2	2
15	M	74	34	Proctitis	+	−	−	−	−	−	3	1	2	Nonresponse	2	1	0	1	1
16	F	35	18	Pancolitis	+	−	−	−	Biofermin	+	5	8	3	Nonresponse	4	2	6	1	2
17	F	20	5	Pancolitis	−	−	Azathioprine	−	−	−	6	8	2	Nonresponse	4	4	5	2	3
18	F	20	3	Pancolitis	+	−	−	−	Bio-Three	+	4	3	1	Nonresponse	3	2	1	1	2
19	F	39	5	Pancolitis	+	+	−	−	Lac-B	+	4	3	1	Nonresponse	3	2	2	1	1
20	M	51	31	Pancolitis	+	−	−	−	−	−	3	3	0	Nonresponse	2	1	2	1	1

Biofermin, *Bifidobacterium bifidum*; Lac-B, *Bifidobacterium longum*+*Bifidobacterium infantis*; Bio-Three, *Streptococcus faecalis*+*Clostridium butyricum*+*Bacillus mesentericus*; Miya-BM, Clostridium butyricum; FMT, fecal microbiota transplantation

**Table2:** Clinical characteristics and outcomes after fecal microbiota transplantation in patients with Crohn’s disease

#	Sex	Age (y)	Montreal classification	CDAI					C-reactive protein (mg/dL)			Hemoglobin (g/dL)		
				preFMT	postFMT		Δ	Outcome	preFMT	postFMT		preFMT	postFMT	
					2W	8W				2W	8W		2W	8W
1	M	39	A2L1B2p	306	201	174	132	Clinical response	0.035	0.011	0.010	8.7	9.3	9.6
2	M	22	A2L3B2p	243	150	160	83	Clinical response	3.260	1.340	5.980	7.1	9.4	10.7
3	F	17	A1L3B1p	175	129	84	91	Clinical remission	0.039	0.009	0.016	9.8	10.0	10.4
4	F	37	A2L3B3p	182	293	182	0	Nonresponse	0.153	0.500	0.331	10.5	10.7	11.2

#1, previous ileocecal resection, treatment with an elemental diet, 5-aminosalicylates, azathioprine, and infliximab (10 mg/kg) #2 , no previous laparotomy, treatment with an elemental diet, 5-aminosalicylates, azathioprine, infliximab (10 mg/kg), Miya-BM, and Lac-B #3, no previous laparotomy, treatment with an elemental diet, 5-aminosalicylates, mercaptopurine, corticosteroid, infliximab (10 mg/kg), and Bio-Three #4, previous ileocecal resection, treatment with 5-aminosalicylates, azathioprine, and adalimumab CDAI, Crohn’s Disease Activity Index; FMT, fecal microbiota transplantation

**Table3:** Clinical characteristics and outcomes after fecal microbiota transplantation in patients with recurrent *Clostridioides difficile* infection

#	Sex	Age (y)	Diarrhea					Toxin A/B					Outcome
			preFMT	postFMT				preFMT	postFMT				
				2W	8W	24W	48W		2W	8W	24W	48W	
1	F	92	+	−	−	−	−	+	−	−	−	−	Clinical response
2	F	52	+	−	−	−	−	+	−	−	−	−	Clinical response
3	M	50	+	−	−	−	−	+	−	−	−	−	Clinical response
4	M	86	+	ND	−	ND	ND	+	ND	−	ND	ND	Clinical response

#1, previous antibiotic treatment with metronidazole, vancomycin, and probiotics (miya-BM) #2, previous antibiotic treatment with metronidazole and probiotics (bio-three) #3, previous antibiotic treatment with metronidazole, vancomycin, and probiotics (biofermin and miya-BM) #4, previous antibiotic treatment with metronidazole and probiotics (miya-BM) FMT, fecal microbiota transplantation

**Table4:** Summary of the gut microbial characteristics of each disease

Disease	Lower bacterial abundance than donors	Bacterial counts increased by FMT
Ulcerative colitis	*Fusicatenibacter saccharivorans*	*Blautia obeum*
	*Eubacterium*	*Eubacterium*
		*Dorea formicigenerans*
		*Fusicatenibacter saccharivorans*
Crohn’s disease	*Blautia*	*Collinsella*
	*Dorea*	*Dorea*
	*Eubacterium*	*Eubacterium*
rCDI	*Fusicatenibacter saccharivorans*	*Sellimonas*
	*Clostridium clostridioforme*	*Oscillibacter*
	*Anaerotignum*	*Anaerotignum*
	*Agathobaculum*	*Bacteroides thetaiotaomicron*
	*Blautia caecimuris*	
	*Blautia faecis*	
	*Faecalibacterium*	
	*Sellimonas*	

FMT, fecal microbiota transplantation; rCDI, recurrent *Clostridioides difficile* infection
