# Supplementary material for: Clinical response and changes in the fecal microbiota and metabolite levels after fecal microbiota transplantation in patients with inflammatory bowel disease and recurrent Clostridioides difficile infection
Source: Fujita Med J. 2020 Nov 13;7(3):87–98. doi: 10.20407/fmj.2020-021 (PMC8749495; doi:10.20407/fmj.2020-021)
Supplement: Supplementary file 1 — Supplementary Figures [file fmj-7-087_s001.pdf]

Supplementary Table. Relative abundance of the microbiome in donors and patients' feces (%)

	Donors	Before antibiotic pretreatment and FMT				Japanese volunteers (n = 106)*
		UC patients		CD patients	rCDI patients	
		Responders	Nonresponders			
	(n =	(n = 5)	(n = 15)	(n = 4)	(n = 4)	
Bacteroides	17.63	18.64	8.57	9.57	0.29	11.37
Prevotella	0.62	1.90	0.66	0.04	0.01	2.38
Eubacterium	0.02	0.11	0.02	0.07	0.03	6.55
Eubacterium hallii	1.22	0.03	0.45	0.00	0.00	-
Clostridium	0.14	0.05	2.16	1.35	0.19	2.28
Faecalibacterium	8.70	8.79	4.38	1.81	0.00	5.74
Unclassified *Firmicutes*	n.d.	n.d.	n.d.	n.d.	n.d.	1.51
Fusicatenibacter saccharivorans	3.94	0.41	0.26	0.02	0.00	-
Ruminococcus	0.18	0.11	0.01	0.00	0.00	5.57
Ruminococcus bromii	1.17	0.00	0.00	0.00	2.08	-
Blautia	12.54	10.37	11.46	8.58	0.27	16.69
Alistipes	3.25	0.21	0.45	0.01	0.87	0.67
Bifidobacterium	9.89	11.29	13.48	9.72	10.30	17.93
Roseburia	0.97	0.40	0.33	1.87	0.00	2.15
Coprococcus	0.23	0.00	0.00	0.28	1.08	1.39
Escherichia	n.d.	n.d.	n.d.	n.d.	n.d.	0.52
Parabacteroides	1.78	1.82	2.42	1.02	0.49	2.02
Dorea	1.82	0.34	0.87	0.13	0.53	2.07
Dialister	0.48	0.53	1.34	1.06	0.05	0.21
Anaerostipes	3.65	2.56	2.28	3.75	0.08	1.96
Streptococcus	2.57	4.71	9.51	3.53	3.55	2.22
Succinatimonas	n.d.	0.00	0.00	n.d.	n.d.	0.01
Butyrivibrio	n.d.	n.d.	n.d.	n.d.	n.d.	0.03
Collinsella	1.83	2.60	1.86	0.12	0.45	3.01
Phascolarctobacterium	0.60	0.31	0.16	0.00	0.00	0.77
Unclassified *Clostridiales*	n.d.	n.d.	n.d.	n.d.	n.d.	1.15
Methanobrevibacter	n.d.	n.d.	n.d.	n.d.	n.d.	0.00004
Akkermansia	0.02	0.00	0.00	0.00	0.00	0.15
Ruminiclostridium	n.d.	n.d.	n.d.	n.d.	n.d.	0.43

FMT, fecal microbiota transplantation; UC, ulcerative colitis; CD, Crohn's disease;
rCDI, recurrent *Clostridioides difficile* infection; n.d., not detected
* Nishijima S, et al. *DNA Res* 2016;23:125-33.
