# Supplementary material for: Clinical response and changes in the fecal microbiota and metabolite levels after fecal microbiota transplantation in patients with inflammatory bowel disease and recurrent Clostridioides difficile infection
Source: Fujita Med J. 2020 Nov 13;7(3):87–98. doi: 10.20407/fmj.2020-021 (PMC8749495; doi:10.20407/fmj.2020-021)

Supplementary Figure 1. Linear discriminant analysis effect size. The analysis was performed using LEfSe version 1.0.7.

A. Responders with ulcerative colitis before fecal microbiota transplantation compared with donors (n = 15).

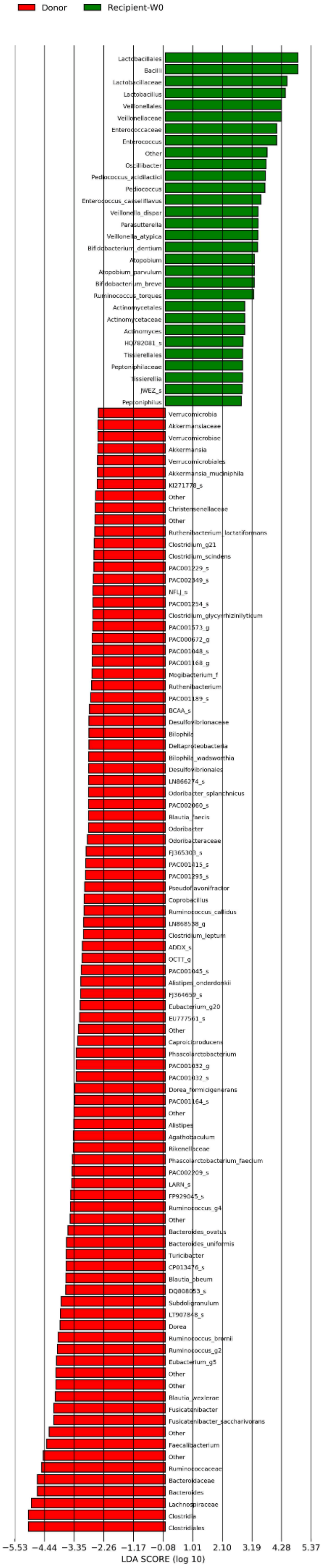

B. Nonresponders with ulcerative colitis 8 weeks after fecal microbiota transplantation compared with donors (n = 15).

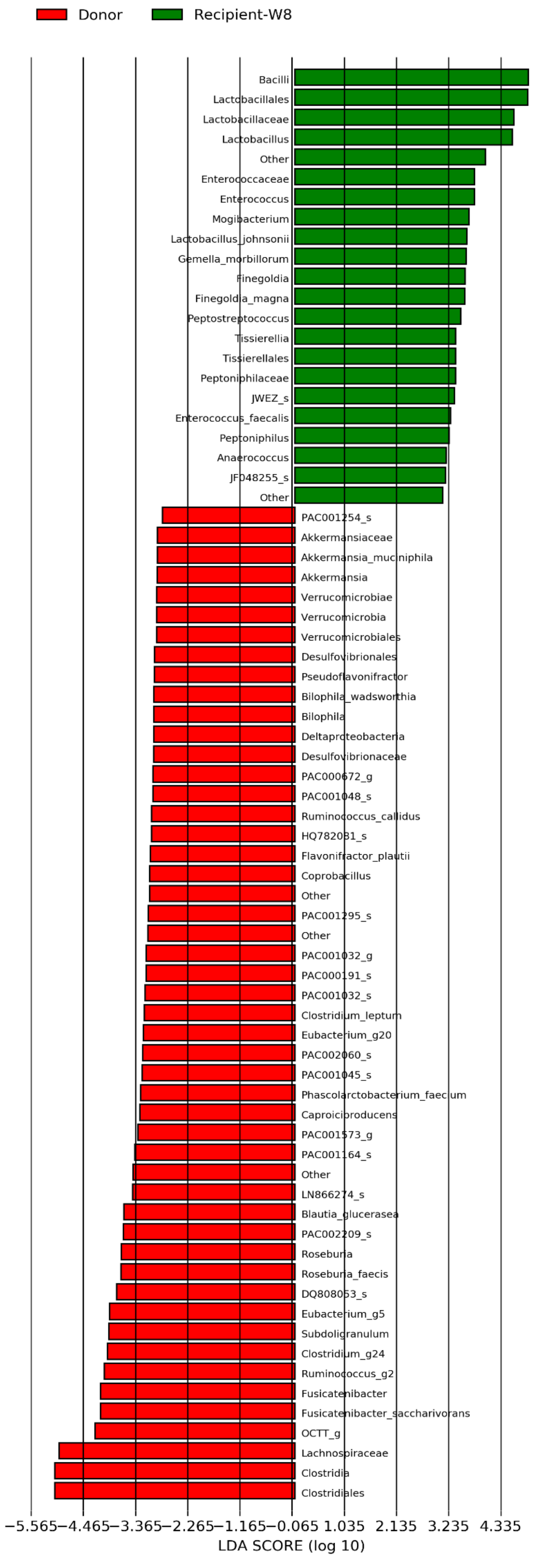

C. Comparison of nonresponders with ulcerative colitis between before and 8 weeks after fecal microbiota transplantation (n = 15).

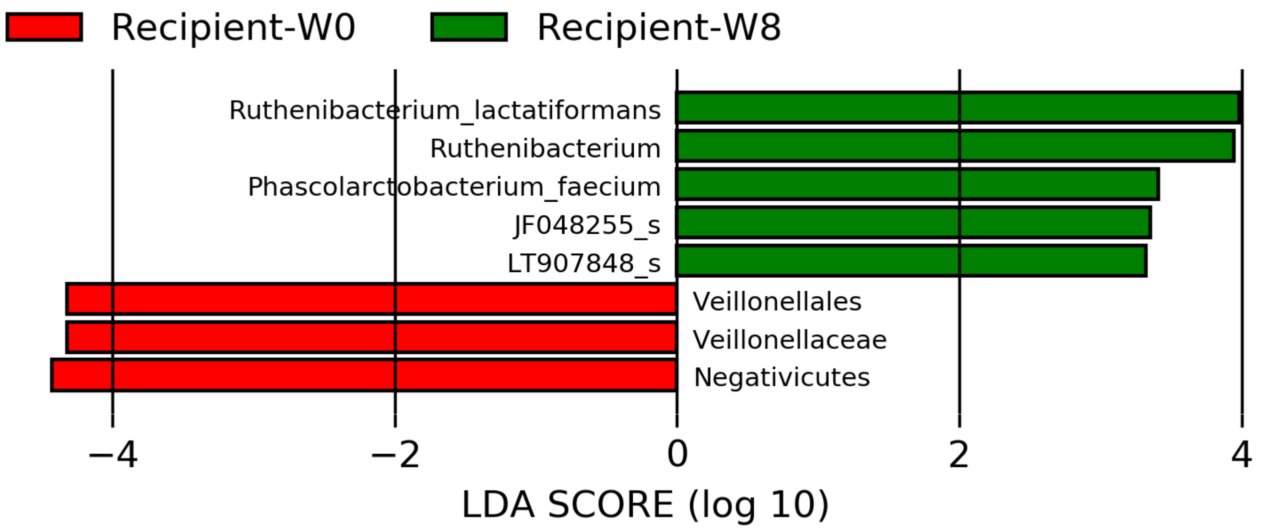

Supplemental Figure 2. Phylogenetic Investigation of Communities by Reconstruction of Unobserved States before antibiotic premedication and fecal microbiota transplantation (FMT) (W0) and 8 weeks after FMT (W8).

A. Crohn's disease.

ANAEROFRUCAT-PWY, homolactic fermentation pathway; FERMENTATION-PWY, mixed acid fermentation pathway; GLYCOLYSIS, glycolysis I (from glucose 6-phosphate) pathway; P164-PWY, anaerobic purine nucleobases degradation I pathway; and PWY-6147, 6-hydroxymethyl-dihydropterin diphosphate biosynthesis I pathway (P = 0.030).

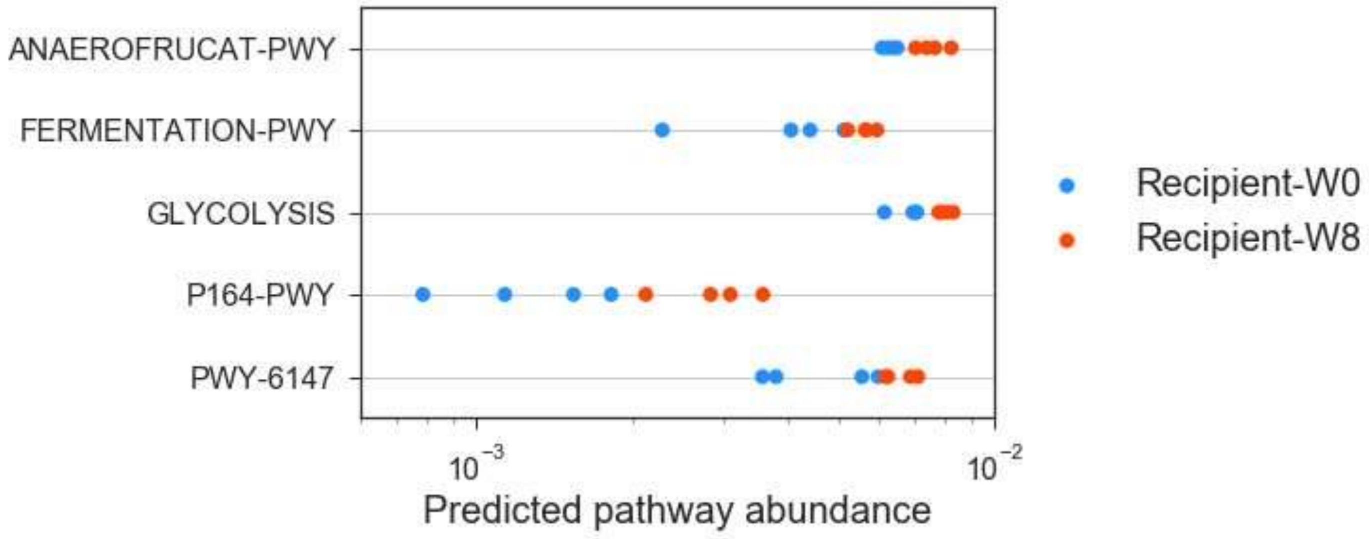

B. Recurrent *Clostridioides difficile* infection.

COBALSYN-PWY, adenosylcobalamin salvage from cobinamide I pathway; COLANSYN-PWY, colanic acid building blocks biosynthesis pathway; GLUCONEO-PWY, gluconeogenesis I pathway; GOLPDLCAT-PWY, superpathway of glycerol degradation to 1,3-propanediol; HOMOSER-METSYN-PWY, L-methionine biosynthesis I pathway; MET-SAM-PWY, superpathway of S-adenosyl-L-methionine biosynthesis; P42-PWY, incomplete reductive TCA cycle pathway; P562-PWY, myo-inositol degradation I pathway; PANTOSYN-PWY, pantothenate and coenzyme A biosynthesis I pathway; PWY-5509, adenosylcobalamin biosynthesis from cobyrinate a,c-diamide I pathway; PWY-5659, GDP-mannose biosynthesis pathway; PWY-5695, urate biosynthesis/inosine 5′ -phosphate degradation pathway; PWY-6269, adenosylcobalamin salvage from cobinamide II pathway; PWY-6969, TCA cycle V (2-oxoglutarate:ferredoxin oxidoreductase) pathway; PWY-7323, superpathway of GDP-mannose-derived O-antigen building blocks biosynthesis; PWY-7539, 6-hydroxymethyl-dihydropterin diphosphate biosynthesis III pathway; and THISYN-PWY, superpathway of thiamin diphosphate biosynthesis I pathway (P=0.030).

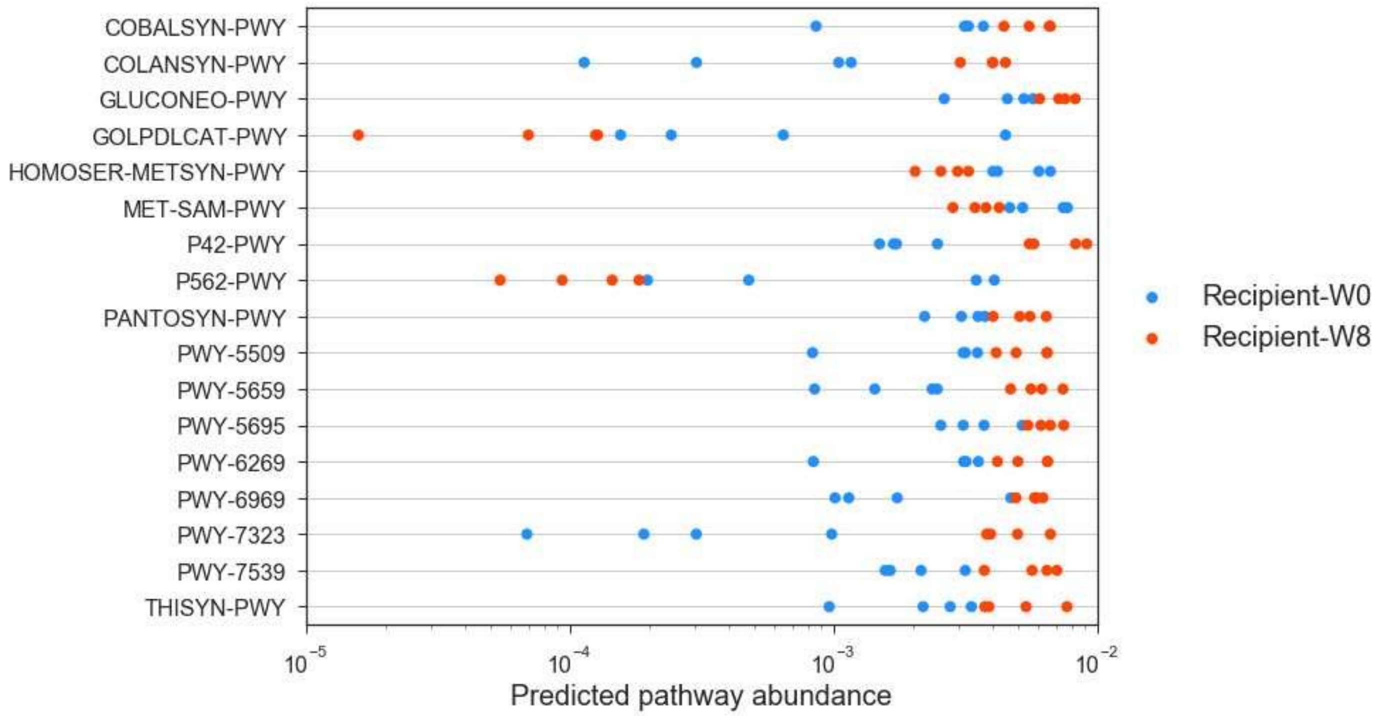

Supplementary Figure 3. Fecal bile acid concentration. Statistical analysis was performed using the Mann–Whitney U test.

A. Ulcerative colitis.

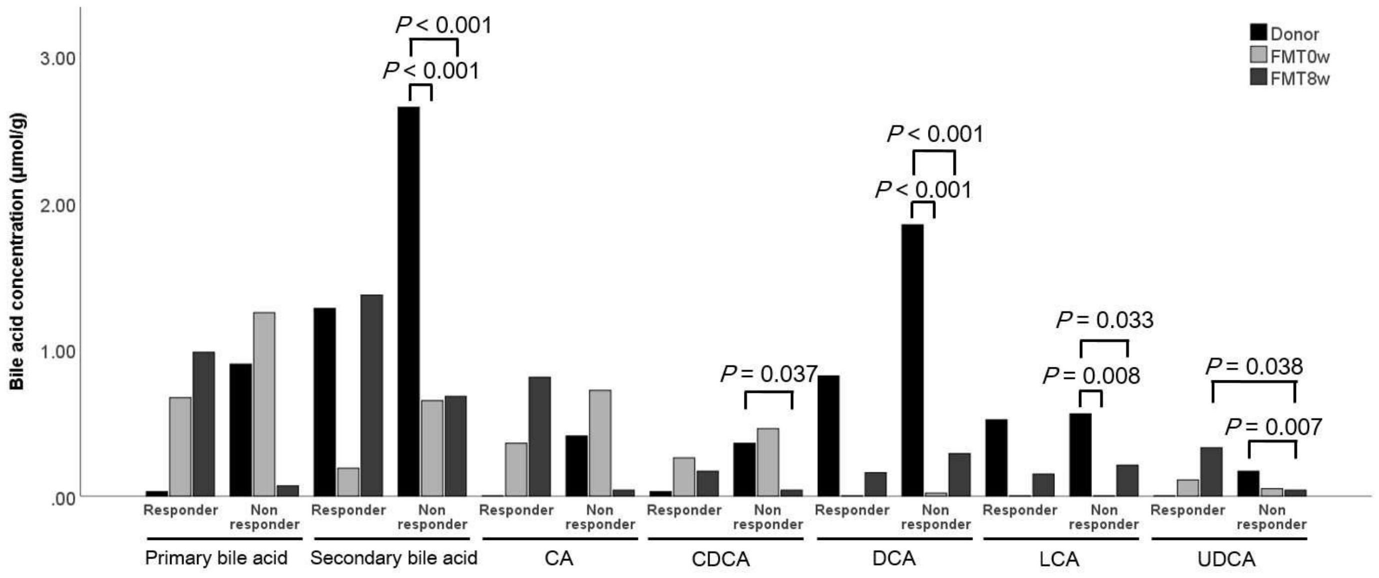

B. Crohn's disease.

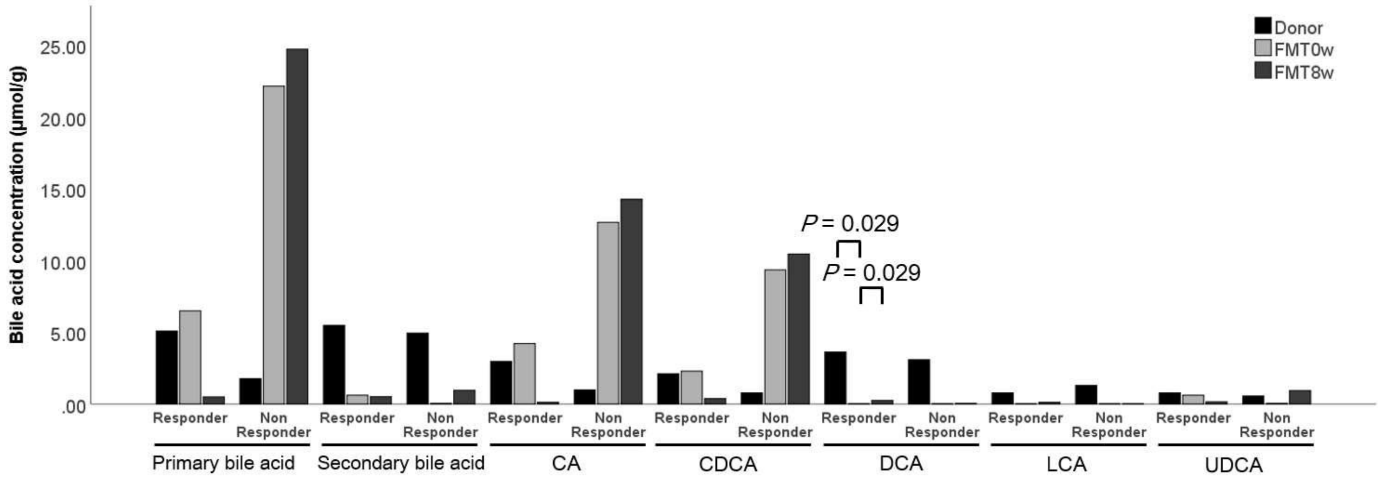

Supplement: Supplementary file 2 — Supplementary Table [file fmj-7-087_s002.pdf]
